# Greener Monolithic Solid Phase Extraction Biosorbent Based on Calcium Cross-Linked Starch Cryogel Composite Graphene Oxide Nanoparticles for Benzo(a)pyrene Analysis

**DOI:** 10.3390/molecules26206163

**Published:** 2021-10-13

**Authors:** Aree Choodum, Nareumon Lamthornkit, Chanita Boonkanon, Tarawee Taweekarn, Kharittha Phatthanawiwat, Wilasinee Sriprom, Wadcharawadee Limsakul, Laemthong Chuenchom, Worawit Wongniramaikul

**Affiliations:** 1 Integrated Science and Technology Research Center, Faculty of Technology and Environment, Phuket Campus, Prince of Songkla University, Kathu, Phuket 83120, Thailand; jar.nareumon@gmail.com (N.L.); chanitanbkn@gmail.com (C.B.); T.tarawee@hotmail.com (T.T.); kharittha.p@gmail.com (K.P.); wilasinee.s@phuket.psu.ac.th (W.S.); wadcharawadee.n@phuket.psu.ac.th (W.L.); worawit.won@phuket.psu.ac.th (W.W.); 2 Center of Excellence for Innovation in Chemistry, Division of Physical Science, Faculty of Science, Hat Yai Campus, Prince of Songkla University, Hat Yai, Songkhla 90110, Thailand; laemthong.c@psu.ac.th

**Keywords:** biocryogel, biosorbent, solid phase extraction, Benzo(a)pyrene, graphene oxide nanoparticle

## Abstract

Benzo(a)pyrene (BaP) has been recognized as a marker for the detection of carcinogenic polycyclic aromatic hydrocarbons. In this work, a novel monolithic solid-phase extraction (SPE) sorbent based on graphene oxide nanoparticles (GO) in starch-based cryogel composite (GO-Cry) was successfully prepared for BaP analysis. Rice flour and tapioca starch (gel precursors) were gelatinized in limewater (cross-linker) under alkaline conditions before addition of GO (filler) that can increase the ability to extract BaP up to 2.6-fold. BaP analysis had a linear range of 10 to 1000 µgL^−1^ with good linearity (*R^2^* = 0.9971) and high sensitivity (4.1 ± 0.1 a.u./(µgL^−1^)). The limit of detection and limit of quantification were 4.21 ± 0.06 and 14.04 ± 0.19 µgL^−1^, respectively, with excellent precision (0.17 to 2.45%RSD). The accuracy in terms of recovery from spiked samples was in the range of 84 to 110% with no significant difference to a C_18_ cartridge. GO-Cry can be reproducibly prepared with 2.8%RSD from 4 lots and can be reused at least 10 times, which not only helps reduce the analysis costs (~0.41USD per analysis), but also reduces the resultant waste to the environment.

## 1. Introduction

Benzo(a)pyrene (BaP) is a nonpolar molecule composed of five fused aromatic rings, and is produced during the incomplete combustion of organic materials. It is one of the most toxic polycyclic aromatic hydrocarbons (PAHs) and has been recognized as a marker for the detection of carcinogenic PAHs [1]. BaP exhibits severe toxic effects on human health even at low concentrations and has been classified as a carcinogen to humans by the International Agency for Research on Cancer (IARC). It has been found in environmental samples of air, soil, and water, and can accumulate gradually in the environment and in humans who are exposed to it as part of everyday life. It is thus considered as an environmental carcinogen that can induce mitochondrial dysfunction and promote metabolic reprogramming [2]. Therefore, the monitoring of BaP contamination levels in the environment is of great significance.

Instrumental analysis methods such as high-performance liquid chromatography (HPLC) [1] and gas chromatography coupled to mass spectrometry (GC-MS) [3,4] are commonly used for BaP analysis to achieve a low detection limit. Hence, sample preparation that can pre-concentrate and pre-extract BaP from sample matrices must be applied before the instrumental analysis to give excellent detection, even at trace levels. Solid-phase extraction (SPE) is one of the most common sample preparation techniques used for this purpose because it is simple, rapid, flexible, emulsion free, convenient, easily automated, and easily coupled with the detection techniques [5]. Various sorbent materials have been reported to achieve good extraction efficiency, sensitivity, selectivity, and reusability, e.g., a cryogel-based molecularly imprinted composite [1], and Fe_3_O_4_/graphene oxide (GO) nanocomposites [6]. GO becomes a desirable sorbent in SPE that can be applied for a wide range of analytes including PAHs due to its excellent adsorption affinity to organic pollutants through non-covalent interactions (π-π stacking, electrostatic interactions and H-bonding) [5,6]. It also has a high specific surface area and is stable in various physicochemical conditions. Recently, GO has been incorporated into a poly(butyl methacrylate-co-ethylene glycol dimethyl methacrylate) monolithic chip for SPE of PAHs [7] and has also been composited with polypyrrole in a polyamide network to enhance the SPE extraction efficiency of parabens [8].

In this work, a novel monolithic graphene oxide nanocomposite biocryogel (GO-Cry) was developed for greener sample preparation of BaP before GC-MS analysis. Native starch was used instead of synthetic polyvinyl alcohol (PVA), which is the most popular polymer that has been used for cryogel preparation both alone and in combination with other polymers [9,10,11], because of its low cost, abundant supply, good processability, biodegradability, and ease of chemical modifications [12,13]. An alternative procedure for greener preparation of macroporous monolithic cryogel using starch was investigated by modifying the procedure to prepare Thai dessert namely “Lod-chong” to prepare the gel precursor from rice flour in the presence of calcium ions from limewater (saturated calcium hydroxide solution), which act as the cross-linker.

## 2. Results and Discussion

### 2.1. Preparation of GO-Cry

**Figure 1:**
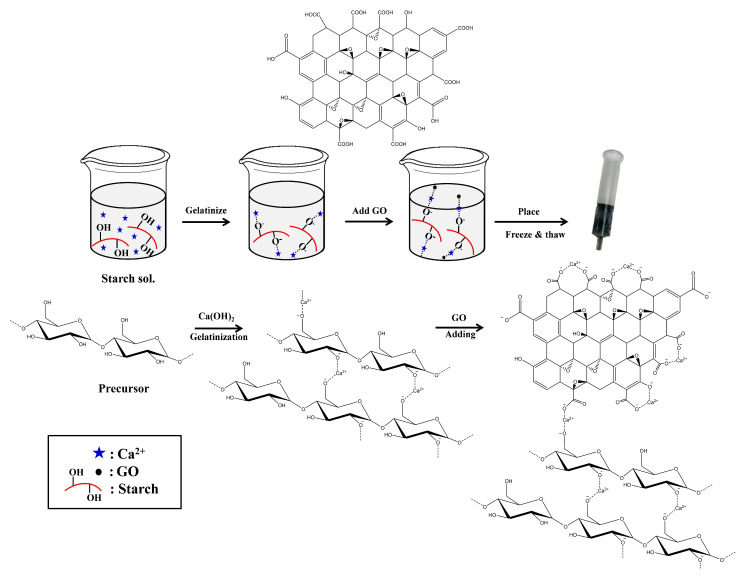
Proposed mechanism for preparation of GO-Cry.

A novel GO-Cry was in situ prepared as a composite of biocryogel (the cryogel without GO: Cry) with graphene oxide nanoparticles (GO). The procedure to prepare this Cry was accidentally found by A. Choodum, who tried to extend the shelf-life of “Lod-chong”, her favorite Thai dessert, by freezing the Lod-chong noodles in the freezer and the noodles started to look like cryogel after defrosting. The procedure to prepare Lod-chong noodles was thus modified to prepare a gel precursor from native starches. Cry was then synthesized using a gel precursor consisting of rice flour, tapioca starch, and arrowroot starch as natural polymers, and limewater (saturated calcium hydroxide solution) as the cross-linker (Figure 1).

Rice flour is the main ingredient to construct a gel structure and to create the texture of the Lod-chong noodles. Since the gel from rice flour is very brittle, tapioca and/or arrowroot starch is commonly blended with rice starch to retain its integrity and to improve its textural properties [14]. When the amount of tapioca starch was increased from 0 to 5 g with 12.5 g rice flour, an interconnected network was obtained in Crys and the compression tolerance increased. This is due to soft swollen granules of tapioca starch, and the leached molecules from tapioca starch granules might bind with the swollen restricted rice flour granules to enhance gel strength [14,15]. Since the physical and mechanical properties of Cry obtained with 3.75 and 5.0 g tapioca starch were similar, the tapioca starch amount of 3.75 g was selected as the optimum value. Arrowroot starch is a common food thickener that may be added to Lod-chong recipe, instead of, or together with, tapioca starch, to increase the viscosity and to decrease retrogradation [16]. However, the results showed that increasing the amount of arrowroot starch from 0 to 5 g in rice flour and tapioca starch provided similar physical and mechanical properties to Cry. It was thus omitted from the recipe in this work. The rice flour (12.5 g) and tapioca starch (3.75 g) were dispersed in limewater and heated for gelatinization. Since the starches were gelatinized in a saturated calcium hydroxide solution that is alkaline, hydroxyl groups of the starches were oxidized, making sites for ionic cross-linking with the Ca^2+^ dissociated from Ca(OH)_2_, and tightening the starch chain [17]. A change of the amount of limewater from 90 to 120 mL provided an inhomogeneous gel precursor due to not having enough water to swell the starch granules and to access their internal structures for solubilization [18,19]. Although homogeneous gels were obtained with 130 to 150 mL limewater, 130 mL was selected to minimize the energy needed for heating an excessive and unnecessary amount of water during the gelatinization.

**Figure 2:**
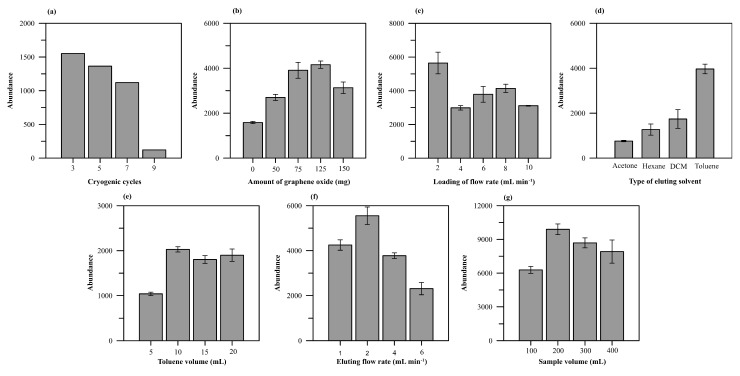
Influence of (**a**) cryogenic cycles, (**b**) amount of GO, (**c**) loading flow rate, (**d**) type of eluting solvent, (**e**) toluene volume, (**f**) eluting flow rate, and (**g**) sample volume on BaP response.

The gelatinized starch was then frozen and thawed to get the cryogel structure. Crys prepared with 1 and 2 cryogelation cycles were damaged from compression on squeezing the materials, while no damage was observed in Crys prepared with 3 freeze-thaw cycles. Since the non-frozen Ca-starch phase would continue cross-linking during freezing, thin walls were formed in the cryogel structure [20,21,22]. Increasing the number of freeze–thaw cycles might increase the crystallinity of starch, in turn increasing the strength of Crys, as reported for a PVA-based cryogel [23]. Although the cryogels prepared with 5, 7, and 9 freeze–thaw cycles tolerated compression, their ability to adsorb BaP was decreased (Figure 2a). This may have been caused by an increase in interconnected macropores in the cryogel, as the growth of ice crystals during freezing could push aside the pore walls [24], thereby giving larger pores with thinner walls. The larger pore size of an adsorbent has a less specific surface area to adsorb an analyte. The analyte was also easily loaded with a higher flow rate, and it was more difficult to retain the analyte in the adsorbent. The material was thus prepared with 3 freeze–thaw cycles as an appropriate choice.

The addition of GO in the range from 0 to 150 mg to the gel precursor (5 g) resulted in the immobilization of GO in Ca-starch phase (Supplementary Material S3a), leading to an increasing BaP response of up to 125 mg and leveling off beyond that (Figure 2b). It is worth noting that BaP could also be extracted using Crys (without GO) due to π-π stacking interactions [25], and in prior reports, starch cross-linked epichlorohydrin gel could adsorb BaP for 1.42 mg/g in 24 h of adsorption time [26]. The added GO can increase the ability to extract BaP by up to 2.6-fold at 125 mg GO, due to the strong π-π interactions [5]. The decrease of BaP response on using 150 mg might be caused by partial agglomeration of GO in the polymer matrix [7,27]. The GO-Cry was thus prepared with 125 mg GO in the gel precursor.

Therefore, GO-Cry was synthesized using the optimum conditions summarized in Table 1.

### 2.2. Characterization of GO-Cry

**Figure 3:**
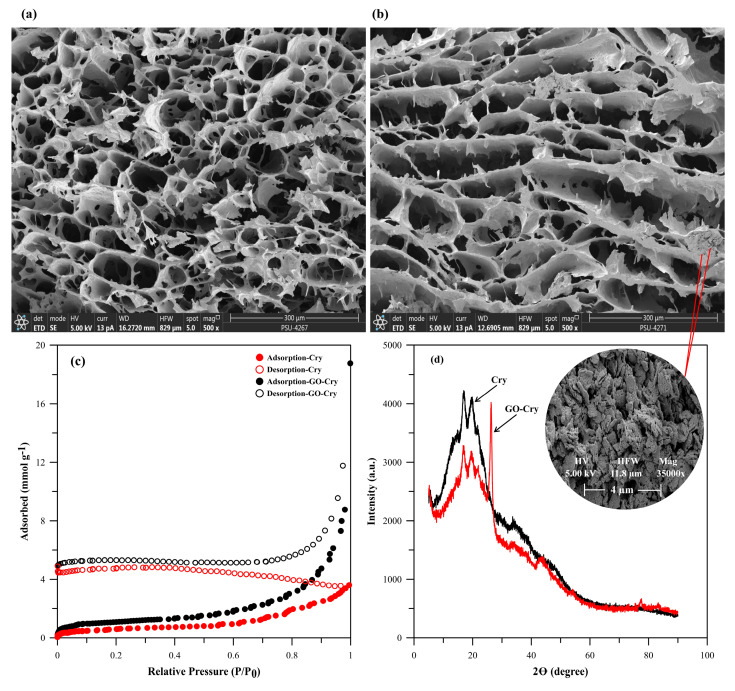
(**a**,**b**) SEM images, (**c**) Nitrogen adsorption isotherms, and (**d**) XRD patterns of Cry and GO-Cry.

**Figure 4:**
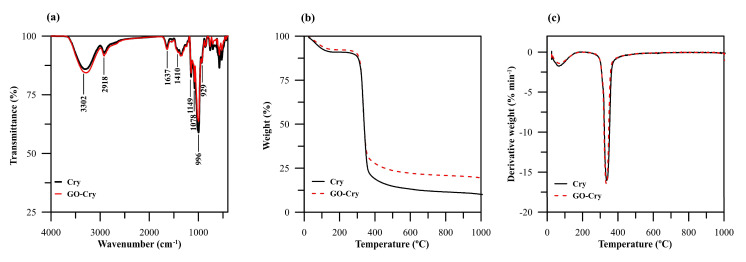
(**a**) FTIR spectra (**b**) TGA (**c**) DTG of Cry and GO-Cry.

The pore size of GO-Cry from SEM images (Figure 3b) was ~47 ± 15 µm, smaller than that of Cry (~53 ± 14 µm, Figure 4a) due to the additional GO in the gel precursor that was immobilized in the Ca-starch phase, leading to thicker pore walls and smaller pores. However, the adsorption average pore diameter of GO-Cry according to BET analysis was 31.0 nm with *S*_BET_ of 3.7411 ± 0.0135 m^2^g^−1^ greater than that of Cry by 176.6% (2.1183 ± 0.0131 m^2^g^−1^ for Cry) due to the large specific surface area of nanoparticle GO, as in a prior report on carboxylate GO incorporated in butylmethacrylate monolith [7]. The total pore volume (V_p_) of GO-Cry (0.029 cm^3^g^−^1) was also larger than that of Cry by 580% (0.005 cm^3^g^−1^) corresponding to the larger estimated porosity from Equation (4) as described in Supplementary Material S1 (38 ± 2% for GO-Cry and 26 ± 1% for Cry). GO-Cry exhibited a type IV adsorption isotherm (Figure 3c), suggesting a mesoporous structure [7,28,29], while Cry showed a type III isotherm, suggesting a macroporous material [30]. GO-Cry had H3 type hysteresis, indicating that the pore network consisted of macropores which are not completely filled with pore condensate [28] and may contribute to the Cry structure within GO-Cry. These results thus indicate a wide distribution of pore sizes in GO-Cry due to being a composite of nanomaterial GO and macroporous Cry. The open hysteresis loops for both Cry and GO-Cry could be attributed to their non-rigid structures that may deform (swell) during adsorption or pore filling and/or the affinity of nitrogen in the material caused by the heterogeneity of the surfaces [30].

The XRD patterns of the GO-Cry (Figure 3d) displayed a strong peak at 2θ ~26.4° for d-spacing of 0.33, indicating this as the characteristic (d002) peak of graphite [31], together with peaks at 15°, 17°, 20°, and 22° indicating double helices of B-type crystalline structure from starch [17]. Since the characteristic (d001) reflection peak of GO that commonly presents 2θ ~11.5°, corresponding to the interplanar distance (d-spacing 0.77 nm) between GO sheets [32,33], disappeared in GO-Cry, this indicates disorder and/or decomposition of the GO sheets during preparation of GO-Cry. The interlayer distance of GO was decreased, maybe because of the interaction of Ca^2+^ in lime water with negative surfaces of GO at a pH higher than its pHpzc (point of zero charge at pH 3.9) [34], causing restacking of the graphene sheets. The crystallinity of Cry increased by 27% on the addition of GO (32.2% for Cry and 41.0% for GO-Cry).

FTIR spectrum of GO-Cry was similar to that of Cry (Figure 4a) due to a small amount of GO in the Cry matrix (2.44%GO in GO-Cry) as well as the disorder or decomposition of GO during preparation. The C=O stretching of COOH groups situated at edges of GO sheets, commonly observed at 1726 cm^−1^ [33], is absent from the GO-Cry spectrum, possibly because of decomposition of the GO sheets and/or the small amount of GO in GO-Cry. For Cry, Ca^2+^ interacted with the OH groups of starch molecules by physical cross-linking via Van der Waals interactions, and no new chemical bonds were observed in the FTIR spectrum [35,36]. The interaction between Ca^2+^ and COOH groups of GO sheets in GO-Cry may be similar and no new chemical bonds were observed. A large band observed at 3302 cm^−1^ was assigned to O–H stretching in the starch molecules [37,38] rather than O–H in the GO molecules. Absorption peaks appeared at 2918 cm^−1^ for the C–H stretching of starch, while the peaks at 1149 and 929 cm^−1^ were assigned to C–O–C vibrations in glycosidic linkages [37,38]. The adsorption peak at 1410 cm^−1^ was assigned to C–H symmetrical scissoring of CH_2_OH moiety [34], while the peaks at 1078 cm^−1^ to 996 cm^−1^ were for the vibrations of C–O bonding in amylopectin and C–OH bending, respectively [37,38].

The thermal stability of GO-Cry and Cry was also investigated and it was found that GO-Cry displayed similar decomposition stages to Cry (Figure 4b). Cry experiences about 9.1% weight loss below 200 °C and 7.7% for GO-Cry as a result of the evaporation of adsorbed water. Both materials then lost their weight by another 65.5% and 59.3% between 300 and 400 °C due to the decomposition of starch [39]. The residual 11.9% and 21.4% weight for Cry and GO-Cry remained after 500 °C, indicating incomplete degradation of the composites. These results indicate that GO in the GO-Cry contributed to better thermal stability than that of Cry. The differential thermogravimetric analysis (DTG) peak of both materials at ~330 °C was from the decomposition of starch (Figure 4c). Since GO has been reported to lose its weight by about 15% below 200 °C with another 20% mass loss between 200 and 250 °C due to the decomposition of oxygen-functional groups at the GO surface [33], the composite with Cry (GO-Cry) showed a higher thermal stability than that of GO due to the interactions between GO and Ca^2+^, as well as between GO and polymeric chains [31].

### 2.3. Optimization of SPE Conditions

The flow rate for sample loading was optimized among the choices 2, 4, 6, 8, and 10 mLmin^−1^ to obtain the fastest flow rate that provides the highest extraction efficiency. The results showed that the response of BaP decreased with the increasing flow rate (Figure 3c) as a high flow rate would not allow BaP to be completely adsorbed by the GO-Cry sorbent. A sample flow rate <2 mLmin^−1^ has been reported to provide similar results to 2 mLmin^−1^ with a longer extraction time [39]. Therefore, the sample flow rate of 2 mLmin^−1^ was used for sample loading.

An eluting solvent was also investigated in order to select a suitable solvent with the highest eluting efficiency. Several solvents with different polarities were investigated, including acetone, dichloromethane, toluene, and hexane. The highest BaP response was obtained from toluene, with polarity index of 2.4 (Figure 2d), while dichloromethane and acetone, which both have higher polarity indexes than toluene (5.1 and 3.1, respectively), provided lower eluting efficiencies. Hexane, with a polarity index of 0.1, also showed a lower BaP response than toluene, but a higher one than acetone. Toluene was thus selected as the eluting solvent. The eluting volume of toluene was then investigated in the range of 5 to 20 mL. It was found that increasing the toluene volume from 5 to 10 mL increased the response of BaP and this remained constant with a further increase of the volume to 15 and 20 mL (Figure 2e). The volume 10 mL was thus selected. Increasing the eluting flow rate from 1 to 2 mLmin^−1^ increased the BaP response, while further increasing the flow rate to 4 and 6 mLmin^−1^ decreased the BaP response (Figure 2f) since a fast eluting flow rate would not give enough time for the analytes to be completely eluted from the sorbent. Adsorbed BaP on GO-Cry was thus eluted using 10 mL toluene with the flow rate of 2 mLmin^−1^.

Sample volume of 100 to 400 mL was also investigated and it was found that increasing the sample volume from 100 to 200 mL increased the BaP response (Figure 2g) due to the larger amount of BaP passing through and the consequently larger amount adsorbed on the sorbent. However, a further increase from 300 and 400 mL decreased the response due to the limited adsorption sites on the sorbent.

Therefore, a 200 mL water sample was loaded to the SPE cartridge containing GO-Cry as the sorbent, with a flow rate of 2 mLmin^−1^. The sorbent was then washed with ultrapure water (10 mL) before drying. The adsorbed BaP was then eluted with 10 mL toluene with a 2 mLmin^−1^ flow rate. The eluent was then evaporated to dryness and the residue reconstituted with 1 mL toluene before analyzing with the GC-MS.

### 2.4. Method Validation

**Figure 5:**
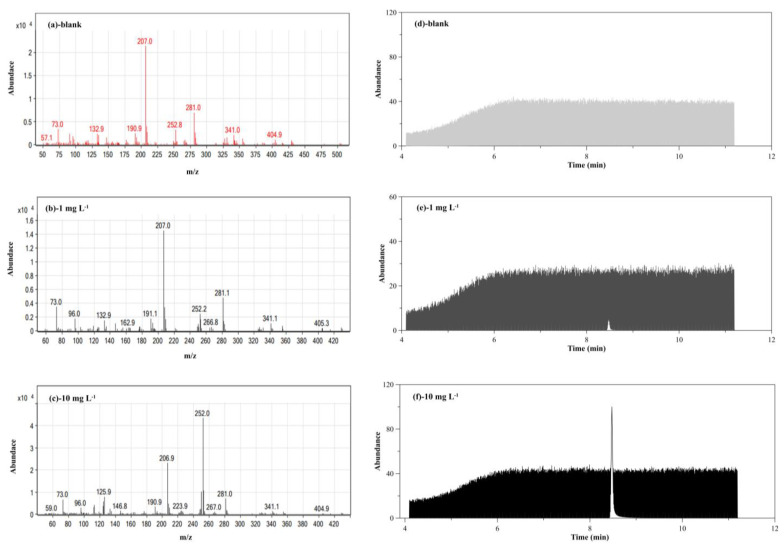
(**a**–**c**) Mass spectra, and (**d**–**f**) chromatograms of BaP at optimum conditions.

Quantification of BaP was performed in an SIM mode at optimum conditions (Figure 5). The linearity, limit of detection (LOD) and limit of quantification (LOQ) based on a standard method [39] were investigated using the optimum conditions. The linear range was found to be from 10 to 1000 µgL^−1^ with a good linearity (*R*^2^ = 0.9971). The limit of detection and limit of quantification based on the IUPAC method (LOD=3SD_Blank_/S and LOQ = 10SD_Blank_/S where SD is the standard deviation of 10 blank analysis and S is the slope of calibration graph) [40] were 4.21 ± 0.06 and 14.04 ± 0.19 µgL^−1^, respectively with excellent precision in terms of relative standard deviations (0.17 to 2.45%RSD).

### 2.5. Analysis of Real Samples

GO-Crys were applied as SPE sorbents for extraction of BaP from sea and surface water samples in Phuket, Thailand. Influence of sea water matrixes was firstly investigated using the sea water collected from the Andaman Sea far away from human activities (2 km from Lone and He Island). BaP was spiked to the sea water and was compared with ultrapure water sample (0.5 mgL^−1^). The BaP response of spiked sea water samples (1494 ± 49 a.u.) did not significantly differ from those in ultrapure water (1496 ± 52 a.u.) at a 95% confidence level (calculated t-value = 1.215, critical t-value = 2.776 at degrees of freedom = 4) with a high recovery (86.4 ± 0.3%).

The BaP was found in water samples collected in December 2019 at concentrations of less than LOQ to 36 ± 4 µgL^−1^, except for sampling point C4 in Chalong Bay, for which BaP could not be detected (Table 2). The highest concentration of BaP (36 ± 4 µgL^−1^) at sampling point C3 near Chalong Pier may be contributed to by incomplete combustion of fuel in crowded yachts and boats, while C1 at a long-tail boat stop showed a lower concentration (10 ± 3 µgL^−1^). The quantified concentrations exceeding BaP solubility in water (1.62 µgL^−1^ at 25 °C) might be attributed to BaP bound to dissolved organic matter (DOM) in water samples. Since SPE with a non-polar eluting solvent (e.g., dichloromethane) can be used to extract PAHs bound to DOM [41], both freely dissolved BaP and DOM-bound BaP could be quantified in this work. In addition, alkaline hydrolysis of organic matter [41] due to Ca(OH)_2_ in the cryogel structure may also help the extraction of BaP bound to DOM. Lower concentrations of BaP were found in all samples collected in October 2020, due to the impact of the COVID-19 pandemic that decreased by 100% the tourism in Phuket, while there were at least 10 million tourists in 2019.

The accuracy of the developed method was evaluated by spiking the water samples with BaP at 50 and 100 µgL^−1^ and the recoveries were in the range of 84 to 110% with 2.04 to 9.75%RSD, within the acceptable range as recommended by the AOAC (70 to 120%) [39] and USEPA (80 to 120% [42]). These good accuracy results indicate that BaP could be quantified by the developed method. SPE extraction using GO-Cry as the sorbent could extract and pre-concentrate BaP from water samples, while some possible interferences can be eliminated in this step. Subsequently, co-eluted substances with BaP from SPE could be separated from BaP by the GC-MS, leading to highly accurate results.

### 2.6. Reproducibility and Reusability

**Figure 6:**
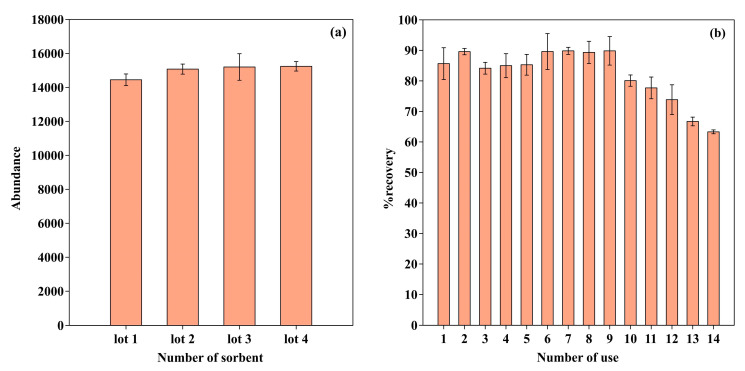
(**a**) Reproducibility and (**b**) reusability of GO-Cry.

The reproducibility in preparation of GO-Cry was investigated in terms of lot-to-lot reproducibility [39]. GO-Cry was prepared in 4 different lots under the same conditions and were applied to BaP extraction and analysis at optimum conditions. The %RSD of BaP response from the 4 lots was 2.8%RSD (Figure 6a), indicating good reproducibility for GO-Cry preparation.

The reusability of GO-Cry for extraction of BaP from water samples was also investigated using BaP spiked samples (0.5 mgL^−1^). The sorbents were washed with 5 mL toluene to investigate the carryover effects after use. It was found that GO-Cry could be used as the SPE sorbent to extract BaP for at least 10 cycles (Figure 6b) without loss of extraction efficiency (80 ± 2%recovery for the 10th cycles), indicating a good stability of the sorbent. The decreasing BaP recovery after 10 cycles might be caused by damage to the Cry network as small black pieces were observed during sample loading. However, GO-Cry showed similar results to the GO composite cryogel prepared from commonly used polyvinyl alcohol that can be re-used for 10 cycles [39], but showed superior performance to the conventional particle-packed SPE sorbent that cannot be reused because of difficulties in removing the adsorbed interferences.

The extraction efficiency of GO-Crys in the extraction of BaP was also compared with conventional particle-packed SPE sorbents (Vertipak C_18_) (Supplementary Material S3b) using spiked water samples. The recovery of 86 ± 5%, with good precision (%RSD < 2.41%), was obtained with no significant difference from the C_18_ cartridge (88 ± 3%recovery, %RSD < 3.30%) at the 95% confidence level (calculated t-value = 0.163, critical t-value = 2.776 at degrees of freedom = 4). Although the cost of GO-Cry was 135 THB (4.1 USD), it can be re-used for 10 cycles, so the cost per analysis is 13.5 THB (0.41USD) compared with 110 THB (3.1USD) per analysis for a conventional C_18_ cartridge. Thus, GO-Cry is a cost effective SPE sorbent with good extraction efficiency, similar to commercial SPE sorbents. The analytical performance of GO-Cry was compared with other reported methods, as summarized in Table 3. The LOD of the method was comparable with the other methods, with a smaller number of active sorbents. GO-Cry can also be reused at least 10 times, which not only helps to reduce the analysis costs, but also reduces the resultant waste to the environment.

## 3. Materials and Methods

### 3.1. Materials

Rice flour (Erawan Brand, Nakhon Pathom, Thailand), tapioca starch (Jaydee Brand, Nakhon Pathom, Thailand), and red lime (no brand, food grade) were purchased from a local supermarket in Phuket, Thailand. The amylose contents of both starches were analyzed using the referenced Thai agriculture standard TAS 4000-2003, and they were 23% and 27%, respectively. GO (15–20 sheets, 4–10% edge oxidized) and BaP were purchased from Sigma-Aldrich (St. Louis, MO, USA), while toluene was from Fisher Scientific (Loughborough, UK). Ethanol (95%, commercial grade) was purchased from S.N.P. Scientific Co. Ltd. (Bangkok, Thailand). Ultrapure water was obtained from a Water Purification System (Merck, Darmstadt, Germany).

### 3.2. Preparation of GO-Cry

The GO-Cry was in situ synthesized in a conventional SPE cartridge by modifying the procedure to prepare famous Thai dessert “Lod-chong” coupled with a conventional freeze and thaw technique. Limewater, which is a saturated calcium hydroxide solution, was prepared for the preparation of starch gel precursor by dissolving red lime in ultrapure water (2.5 gL^−1^) and was kept for at least 3 days before taking the upper clear supernatant solution. Rice flour (12.5 g), tapioca starch (0, 1.25, 2.50, 3.75, 5.00 g), and arrowroot flour (0, 1.25, 2.50, 3.75, 5.00 g) were dispersed in limewater (90, 100, 110, 120, 130, and 150 mL). The mixture was then gradually heated from 90 to 200 °C for 1.5 h to get completely gelatinized starch. GO (0 to 150 mg) was then added in starch gel (6 g) and further mixed to obtain a homogenous mixture, before cooling the mixture under continued stirring for 5 min. The mixture was then poured into a 6 mL empty SPE cartridge, filling it tightly, before placing it in a freezer at −20 °C for 24 h. The resultant material was then thawed at room temperature before repeated freeze and thaw cycles for an appropriate number of times (3, 5, 7, 9 cycles). It was then ready to connect with the SPE manifold.

### 3.3. Characterization of GO-Cry

The morphology of GO-Cry was investigated using field emission scanning electron microscopy (FE-SEM; FEI). Its functional groups were analyzed with Fourier transform infrared spectroscopy (FT-IR; Bruker, Berlin, Germany) using KBr pellet at 4000–600 cm^−1^. X-ray diffractometer (Empyrean, PANalytical, Almelo, Netherlands) was used to investigate XRD patterns using monochromatic Cu-Kα radiation. A high throughput surface area and porosity analyzer (ASAP2460, Micromeritics, Norcross, GA, USA) was used to determine the nitrogen adsorption/desorption isotherm. GO-Cry was degassed at 105 °C for 30 min to remove physically absorbed gases from the sample surfaces before analysis. The specific surface area (S_BET_) was calculated using the Brunauer–Emmet–Teller (BET) method, while the pore volume was obtained from an adsorption branch using the Barrett, Joyner and Halenda (BJH) method. The average pore diameter was calculated from the pore volume and S_BET_.

The swelling ratio, water uptake capacity, water retention, and porosity of GO-Cry were investigated and they are discussed in the Supplementary Material S1.

### 3.4. Analysis of BaP using GC-MS

The analysis of BaP was performed using an Agilent 7890A gas chromatograph equipped with Agilent 5977B mass spectrometer and Agilent 7693 autosampler (Agilent Technologies Inc., Santa Clara, CA, USA). The separation conditions using HP-5MS capillary column (30 m length × 0.25 mm id × 0.25 μm film thickness) were optimized by changing one parameter whilst keeping the other parameters constant using the starting conditions reported in the literature [14]. Once an optimum for a parameter was obtained, it was used to optimize the next parameter, and the final optimum conditions were used throughout the work as described in Supplementary Material S2. Helium (99.9995%) was used as a carrier gas with an optimum flow rate of 1 mLmin^−1^. The injection port was set at 300 °C using a split ratio 30: 1 with 1 μL injection volume. The oven temperature program was set at 160 °C for 1 min, raised to 290 °C at a rate of 25 °Cmin^−1^ and maintained at this temperature for 6 min resulting in 11.30 min total run time. The solvent delay was 4 min, while the MS transfer line temperature was set to 250 °C, with setpoint 230 °C for the ion source (electron impact at ionization energy 70 eV), and 150 °C for the quadrupole mass analyzer temperature. Full scan spectrum analysis was used to obtain the full mass spectra of BaP, while a selected ion monitoring (SIM) mode was used for BaP quantification. An abundance of quantifier ion (m/z 252) at a BaP retention time (8.48 min) was used to establish its calibration graph, while m/z 250, and 252 were used as qualifier ions [43,47].

### 3.5. Solid Phase Extraction (SPE) for BaP Analysis

For the SPE experiments, 990 mL of ultrapure water samples were spiked with a stock solution of BaP in toluene (10 mL, 1000 mgL^−1^) in order to obtain a concentration of 10 mgL^−1^. Although the prepared concentrations exceeded the BaP solubility in water (1.62 µgL^−1^ at 25 °C), the aqueous samples were free from BaP crystals due to cosolvent effect. The GO-Crys in-situ synthesized in 6.0 mL polypropylene cartridge was first connected to the SPE manifold (12-Port Vacuum Manifold, Vertical^®^). It was then conditioned with 5.0 mL ultrapure water before loading a sample for the appropriate volume (100, 200, 300, and 400 mL) with optimized flow rate (2, 4, 6, 8, and 10 mLmin^−1^). The sorbent was then washed with 10 mL ultrapure water before drying for 3 min. The adsorbed BaP were then eluted with appropriate eluting solvent (acetone, hexane, dichloromethane, or toluene) for optimized volume (5, 10, 15, 20 mL) with optimized flow rate (1, 2, 4, 6 mLmin^−1^). The eluent was then evaporated to dryness by nitrogen purging, and the sample was reconstituted with 1 mL toluene before analyzing with the GC-MS.

### 3.6. Real Sample Analysis

**Figure 7:**
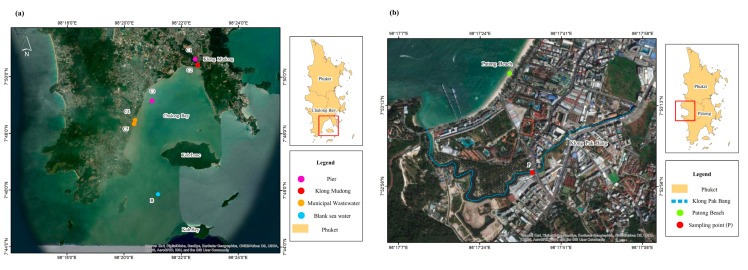
Sampling points for water samples at (**a**) Chalong Bay, and (**b**) Pak Bang Canal in Patong, Phuket, Thailand.

GO-Crys were applied as the SPE sorbent for extraction of BaP from sea and surface water in Phuket, Thailand (Figure 7). Five sea water samples were collected from Chalong Bay, which is a large bay with the pier that is the principal boat anchorage, in December 2019 and October 2020. One surface water sample was also taken from Pak Bang Canal in Patong in the same period. Three replicates of all samples (200 mL) were prepared using GO-Crys at optimum conditions before analyzing with GC-MS.

## 4. Conclusions

A novel GO-Cry SPE sorbent was in situ prepared as a composite of biocryogel with graphene oxide (GO). Rice flour and tapioca starch were gelatinized in limewater under alkaline conditions, leading to ionization of their hydroxyl groups, which enabled ionic cross-links with the Ca^2+^ dissociated from limewater, tightening the starch chains. GO was then added to the mixture in order to increase the ability to extract BaP by up to 2.6-fold, due to the strong π-π interactions, although GO sheets decomposed to graphite during preparation due to the interactions of Ca^2+^ in the cryogel matrix with carboxyl groups in GO. GO-Cry can be applied as an SPE sorbent for extraction of BaP from water samples, with a linear range from 10 to 1000 µgL^−1^ with good linearity (*R^2^* = 0.9971) and high sensitivity (4.1 ± 0.1 a.u./(µgL^−1^)). The limit of detection and the limit of quantification based on IUPAC methods were 4.21 ± 0.06 and 14.04 ± 0.19 µgL^−1^, respectively with excellent precision in terms of relative standard deviations (0.17 to 2.45%RSD). The accuracy in terms of recovery from spiked samples was in the range of 84 to 110%, with 2.04 to 9.75RSD% and no significant differences in the C_18_ cartridge at 95% confidence level. GO-Cry can also be reused at least 10 times, which not only helps to reduce the analysis costs (~0.41USD per analysis), but also reduces the resultant waste to the environment.

## Figures and Tables

**Table 1:** Optimum conditions to prepare GO-Cry.

Component	Study Value	Optimum Value
Rice flour (g)	12.5	12.5
Tapioca starch (g)	0, 1.25, 2.50, 3.75, 5.00	3.75
Arrowroot starch (g)	0, 1.25, 2.50, 3.75, 5.00	0
Limewater (mL)	90, 100, 110, 120, 130, 150	130
GO (mg)	0, 50, 75, 125, 150	125
Freeze–thaw cycle (cycle)	3, 5, 7, 9	3

**Table 2:** Real sample analysis.

Sample	December 2019			October 2020
	Added (µg L^−1^)	Found (µg L^−1^)	%Recovery	Found (µg L^−1^)
C1	0 50 100	10 ± 3 * 58 ± 2 106 ± 4	0 97 97	ND **
C2	0 50 100	7 ± 0 * 49 ± 3 108 ± 0	0 84 101	ND **
C3	0 50 100	36 ± 4 * 83 ± 3 138 ± 2	0 95 102	28 ± 1 *
C4	0 50 100	ND ** 58 ± 1 95 ± 3	0 110 92	ND **
C5	0 50 100	5 ± 1 * 52 ± 3 89 ± 2	0 94 84	ND **
P	0 50 100	5 ± 1 * 53 ± 3 105 ± 6	0 96 100	ND **

* LOD (4.21 ± 0.06 µgL^−1^) < concentration < LOQ (14.04±0.19 µgL^−1^) ** ND = non detectable.

**Table 3:** Comparison of GO-Cry and other reported methods for the analysis of BaP.

Method	Sample Preparation	Amount of Adsorbent	Sample Volume	LOD (µg L^−1^)	Reusability	Reference
GC/MS	Mega bond elut C18 SPE	5 g	4 mL	0.05	No	[43]
GC/MS	C18 Sep-Pak SPE	500 mg	100 mL	4	No	[44]
GC/MS	C18/Fe_3_O_4_/ µSPE	50 mg	20 mL	5.6	-	[45]
GC/MS	Graphene disk SPE	200 mg	1000 mL	0.013	10	[46]
GC/MS	GO-Cry SPE	25 mg (GO)	200 mL	4.21 ± 0.06	10	This work

## Data Availability

All data are available from the corresponding author on reasonable request.

## Supplementary Materials

The following are available online, Figure S1: (a) Swelling ratio, (b) Water uptake capability, and (c) Water retention of GO-Cry and Cry, Figure S2: Optimization of GC conditions for analysis of BaP (a,b) carrier gas flow rate, (c) column initial temperature, (d) initial holding time, (e) ramp rate, (f) final temperature, (g) final holding time, and (h) inlet temperature, Figure S3: (a) GO-Cry with amounts of GO from 0 to 150 mg from the left-hand side, (b) GO-Cry and conventional C_18_ SPE cartridge [48,49,50,51].
