# Supplementary material for: Greener Monolithic Solid Phase Extraction Biosorbent Based on Calcium Cross-Linked Starch Cryogel Composite Graphene Oxide Nanoparticles for Benzo(a)pyrene Analysis
Source: Molecules. 2021 Oct 13;26(20):6163. doi: 10.3390/molecules26206163 (PMC8539787; doi:10.3390/molecules26206163)
Supplement: Supplementary file 1 [file molecules-26-06163-s001.zip › molecules-1382268-supplementary.pdf]

Greener Monolithic Solid Phase Extraction Biosorbent Based on Calcium Cross-Linked Starch Cryogel Composite Graphene Oxide Nanoparticles for Benzo(a)pyrene Analysis

Aree Choodum [1,*], Nareumon Lamthornkit [1], Chanita Boonkanon [1], Tarawee Taweekarn [1], Kharittha Phatthanawiwat [1], Wilasinee Sriprom [1], Wadcharawadee Limsakul [1], Laemthong Chuenchom [2], and Worawit Wongniramaikul [1]

[1] Integrated Science and Technology Research Center, Faculty of Technology and Environment, Prince of Songkla University, Phuket Campus, Kathu, Phuket 83120 Thailand; jar.nareumon@gmail.com (N,L,); chanitanbkn@gmail.com (C.B.); T.tarawee@hotmail.com (T.T.); kharittha.p@gmail.com (K.P.); wilasinee.s@phuket.psu.ac.th (W.S.); wadcharawadee.n@phuket.psu.ac.th (W.L.); worawit.won@phuket.psu.ac.th (W.W.)

[2] Division of Physical Science and Center of Excellence for Innovation in Chemistry, Faculty of Science, Prince of Songkla University, Hat Yai Campus, Hat Yai, Songkhla 90110 Thailand; laemthong.c@psu.ac.th

* Correspondence: Email address: aree.c@phuket.psu.ac.th; Tel.: +66(0)7627 6481

1. Swelling ratio, water uptake capacity, water retention, and porosity of GO-Cry

The swelling ratio of GO-Crys ($S_{g/g}$) was investigated following prior reports [48,49] by weighing the dried and swollen GO-Crys and applying Equation (1) [48,49]. The thawed GO-Crys was removed from the container before cutting into smaller pieces (1cm length×1 cm diameter) and soaked in 95% ethanol for 24 h before drying in an oven at 100°C until stable weight. Three completely dried GO-Crys with similar weights (W_0 ~ 0.19 g) from different thawed monolithic GO-Crys samples were equilibrated in 30 mL ultrapure water at ambient temperature. The water-adsorbed GO-Cry were weighed after removing the surface excess water with filter paper at 1 to 60 min (W_t).

$$\text{Swelling ratio } (S_{g/g}) = \frac{(W_t - W_0)}{W_0}, \quad (1)$$

The water uptake capacity (%) was calculated using Equation (2) [48,49] where W_e is the weight of swollen GO-Crys at the equilibrium.

$$\text{Water uptake capacity (\%)} = 100 \times \frac{(W_t - W_0)}{(W_e - W_0)} \quad (2)$$

The equilibrated GO-Crys were then put in Petri dishes and re-weighted at certain time intervals. The weights of the cryogels (W_T) were recorded during the course of deswelling until they had reached saturated weight values. The water retention (%) can be calculated using the following Equation (3) [48].

$$\text{Water retention (\%)} = 100 \times \frac{(W_T - W_0)}{(W_e - W_0)} \quad (3)$$

The porosity of GO-Crys (%) was also determined by squeezing the swollen GO-Crys and using Equation (4) [50]. The weight of swollen gel (W_e) was compared to the weight after squeezing (W_q).

$$\text{Porosity (\%)} = 100 \times \frac{(We - Wq)}{We} \quad (4)$$

The swelling ratio of GO-Cry was less than that of Cry and they increased with the time at room temperature and reached the equilibrium at 15 minutes (5.7±0.3 $g_{water}/g_{material}$ for GO-Cry and 7.5±0.2 $g_{water}/g_{material}$ for Cry) (S1a). Since swelling is by expansion of the cryogel network due to the interaction between the polymeric chain networks and water molecules, the additional GO in Cry would increase hydrophobicity of the material leading to decreased interactions with water. The addition of GO in Cry would also make the

network denser and difficult to expand, so the GO-Cry thus has a lesser swelling ratio than Cry. The water would be completely adsorbed in GO-Cry after 15 minutes (S1b) for 567±27% of its dry weight and 752±16% for Cry, although water uptake capability of GO-Cry was less than that of Cry in the beginning (< 10 minutes). This may be caused by the smaller macropores in GO-Cry, which could limit the penetration of water into the network. The water retention ability of GO-Cry was also investigated by reversing the swelling process and it decreased with time (S1c). The Cry lost adsorbed water by ~54% in 4 hours, more than GO-Cry that lost 44%; however both of them lost ~99% of the adsorbed water in 48 hours. The better water retention ability for GO-Cry than for Cry may be due to the mesopores of GO nanoparticle composites with smaller macropores than in Cry, and this could limit the evaporation of water from GO-Cry relative to Cry.

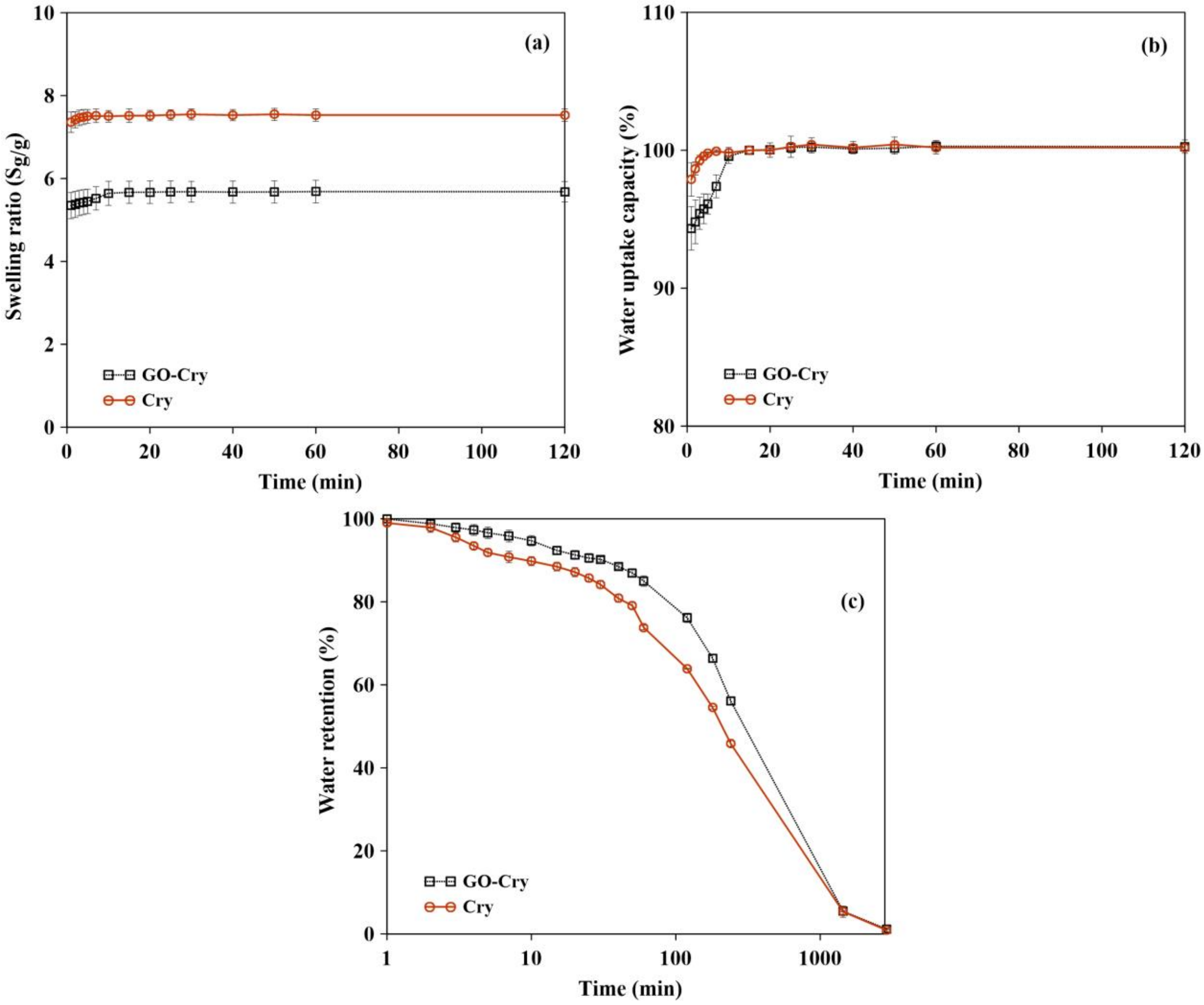

Figure S1. (**a**) Swelling ratio, (**b**) Water uptake capability, and (**c**) Water retention of GO-Cry and Cry.

2. Optimization of GC conditions

The analysis of BaP was performed using an Agilent 7890A gas chromatograph equipped with Agilent 5977B mass spectrometer and Agilent 7963 autosampler (Agilent Technologies Inc., USA, https://www.agilent.com/). The separation GC conditions using HP-5MS capillary column (30 m length × 0.25 mm id × 0.25 μm film thickness) were optimized by changing one parameter whilst keeping the other parameters constant, using the starting conditions reported in the literature [14]. Once an optimum for a parameter was obtained, it was used to optimize the next parameter, and the final optimum conditions were used throughout the work. The solvent delay was 4 min, while the MS transfer line temperature was set to 250°C, with setpoint 230°C for the ion source (electron impact at ionization energy 70 eV), and 150°C for the quadrupole mass analyzer temperature.

Full scan spectrum analysis was used to obtain the full mass spectra of BaP during optimization.

3. Carrier gas flow rate

The van Deemter plot of BaP was done using an ultrahigh purity (99.9995%) helium carrier gas flow rate of 0.5 to 3.0 mL min^{-1} and the plate number (N) was estimated using Equation (5):

$$N = 2\pi(t_R h / A)^2 \quad (5)$$

where t_R is the retention time, h is peak height, and A is peak area of BaP. Although the use of temperature programming could affect the estimated plate number [51], the experiments were performed under the same temperature program, and similar effects were expected. BaP provided the lowest height equivalent to a theoretical plate (HETP) at 2.5 mL min^{-1} (S2a), however its response was the highest at 1.0 mL min^{-1} (S2b). The carrier gas flow rate of 1.0 mL min^{-1} was thus selected as the optimized value to save the amount of gas as well as getting the highest response.

4. Column initial temperature

The initial column temperature was investigated in the range from 140 to 170°C and it was found that the peak areas increased with increasing initial column temperature from 140 to 160°C and then leveled off (S2c). The column initial temperature of 160°C was thus selected.

5. Column initial holding time

The initial holding time was investigated for 0 to 3 minutes. An increase in the peak areas of BaP response was revealed when the holding time was slightly increased from 0 to 2 minutes and then it leveled off (S2d). As the column was kept at a much lower temperature than BaP boiling point (~495°C), holding it for longer may cause some losses [51]. In addition, increasing of holding time resulted in an increase in the retention times of BaP, a hold time of 1 minutes was thus selected.

6. Column ramp rate

The column ramp rate was investigated from 10°C to 30°Cmin^{-1} (S2e). Peak areas of BaP tend to increase with the ramp rate from 10°C to 25°Cmin^{-1} as increasing the ramp rate resulted in increasing of retention temperature that made BaP might be eluted at higher temperature (closer to their boiling points), thus its concentration in mobile phase would be higher and give a larger peak area [51]. However, the peak area decreased at 30°Cmin^{-1} –as a consequence the ramp rate 25°Cmin^{-1} was selected.

7. Final column temperature

The final column temperature was studied in the range from 290 to 320°C and the peak area slightly increased with only little difference observed (S2f). Final column temperature of 290°C was thus selected in order to reduce the total analysis and cool down times.

8. Final column temperature holding time

The final column temperature holding time was investigated from 4 to 12 minutes. The peak area of BaP remained constant from 4 to 6 minutes and leveled off after that (S2g). A final column temperature (290°C) was thus held for 6 min in the analysis of BaP.

9. Inlet temperature

The optimum inlet temperature was investigated from 280 °C to 320 °C. It was found that the peak area of BaP increased with increasing inlet temperature from 280 °C to 310

°C (S2h) due to better vaporization and remained constant at 320 °C. Although the highest response was obtained at 310 °C, the inlet temperature was selected as 300 °C to extend the lifetime of inlet septum that has maximum temperature at 325 °C.

From the results, helium was used as a carrier gas with optimum flow rate of 1 $mLmin^{-1}$. The injection port was set up at 300 °C using split ratio of 30:1 with 1 μL injection volume. The oven temperature program was set at 160 °C for 1 min, ramping to 290 °C at a rate of 25 $°C \times min^{-1}$ and maintaining at this temperature for 6 min for an 11.30 min total run time.

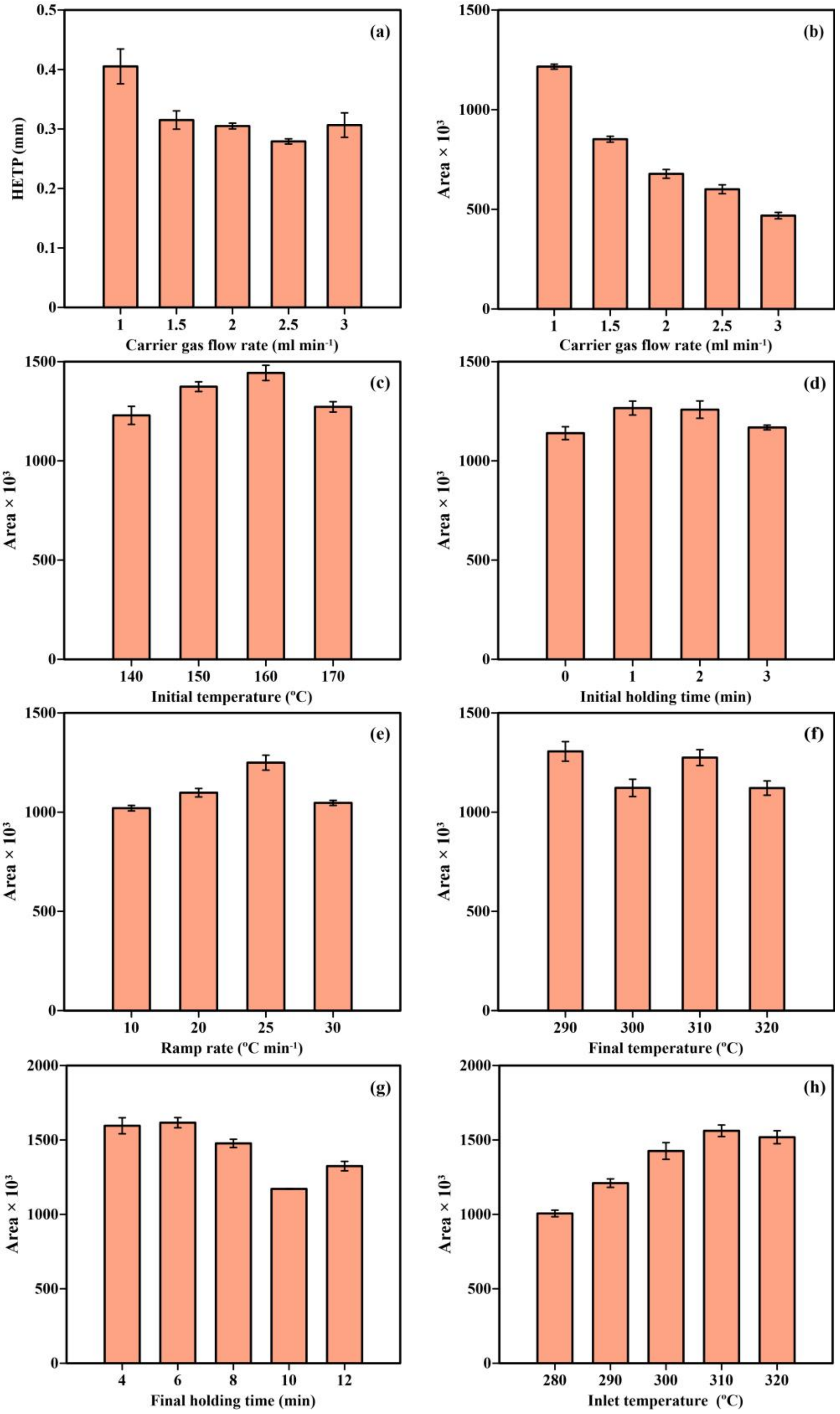

Figure S2. Optimization of GC conditions for analysis of BaP (**a-b**) carrier gas flow rate, (**c**) column initial temperature, (**d**) initial holding time, (**e**) ramp rate, (**f**) final temperature, (**g**) final holding time, and (**h**) inlet temperature.

10. GO-Cry SPE cartridge

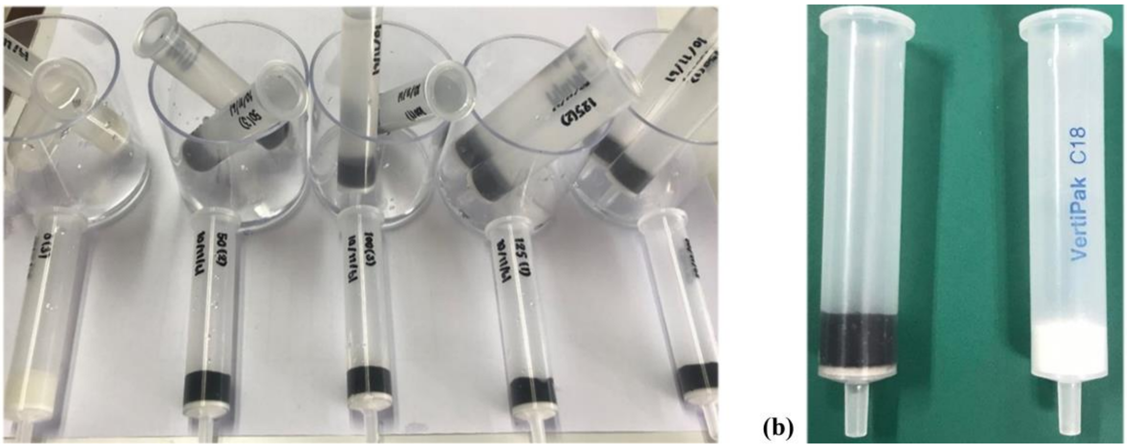

Figure S3. (**a**) GO-Cry with amounts of GO from 0 to 150 mg from the left-hand side, (**b**) GO-Cry and conventional C_{18} SPE cartridge.

References

14. Ledesma, E.; Rendueles, M.; Díaz, M. Spanish smoked meat products: Benzo(a)pyrene (BaP) contamination and moisture. *J. Food Compos. Anal.* **2015**, 37, 87-94.
48. Jayaramudu, T.; Ko, H.U.; Kim, H.C.; Kim, J.W.; Kim, J. Swelling Behavior of Polyacrylamide-Cellulose Nanocrystal Hydrogels: Swelling Kinetics, Temperature, and pH Effects. *Materials* **2019**, *12*, 2080.
49. Xue, W.; Champ, S.; Huglin, M.B.; Lones, T.G.J. Rapid swelling and deswelling in cryogels of crosslinked poly(N-isopropylacrylamide-co-acrylic acid). *Eur. Polym. J.* **2004**, *40*, 467-476.
50. Ertürk, G.; Mattiasson, B. Cryogels-versatile tools in bioseparation. *J. Chromatogr. A* **2014**, *1357*, 24-35.
51. Choodum, A.; Daéid, N.N. Development and validation of an analytical method for hydrocarbon residues using gas chromatography-mass spectrometry. *Anal. Methods* **2011**, *3*, 1136-1142.
